# Overdrive pacing in the acute management of osimertinib-induced ventricular arrhythmias: A case report and literature review

**DOI:** 10.3389/fcvm.2022.934214

**Published:** 2022-09-29

**Authors:** Yanyu Zhang, Xingtong Wang, Yilin Pan, Beibei Du, Kumaraswamy Nanthakumar, Ping Yang

**Affiliations:** ^1^Department of Cardiology, China-Japan Union Hospital of Jilin University, Jilin Provincial Cardiovascular Research Institute, Changchun, China; ^2^Department of Cardiology, Inner Mongolia Autonomous Region Cancer Hospital, Hohhot, China; ^3^National Key Discipline in Hematology, Department of Hematology, The First Hospital of Jilin University, Changchun, China; ^4^The Hull Family Cardiac Fibrillation Management Laboratory, Toronto General Hospital, University Health Network, Toronto, ON, Canada

**Keywords:** osimertinib, QTc interval prolongation, ventricular tachycardia, temporary pacemaker, torsade de pointes (TdPs)

## Abstract

QT interval prolongation and ventricular arrhythmias (VAs) induced by osimertinib, a third-generation epidermal growth factor receptor tyrosine kinase inhibitor, are life-threatening complications. However, no consensus has been achieved regarding their management. Overdrive pacing has been shown to be effective in shortening the QT interval and terminating torsade de pointes (TdP). Here, we report a case of osimertinib-induced QT prolongation accompanied by frequent VAs and TdP. Osimertinib was immediately discontinued after it was identified as the etiology for QT prolongation and VAs. A temporary pacemaker and overdrive pacing were used after other anti-arrhythmia treatments had failed and successfully shortened the QTc interval and terminated VAs. Repeated Holter monitoring at 1 week showed no remaining VAs or TdP, and the pacemaker was removed. Routine electrocardiography (ECG) surveillance was conducted afterward, and three- and 6-month follow-ups showed good recovery and normal ECG results. Vigilance is required for rare vital arrhythmias in patients taking osimertinib, and ECG surveillance should be conducted.

## Introduction

Epidermal growth factor receptor (EGFR) mutation is one of the most common oncogenic drivers in non-small cell lung cancer (NSCLC). Osimertinib, the third-generation EGFR tyrosine kinase inhibitor (TKI), has substantially improved treatment efficacy for NSCLC with EGFR mutations (1). However, while remaining low in incidence, cardiotoxicities related to EGFR-TKIs, such as congestive heart failure, QT interval prolongation, and ventricular arrhythmias (VAs) have become a safety concern (2), as they are life-threatening complications.

No consensus has been achieved for the management of these cardiotoxicities (2, 3). Overdrive pacing, or pacing with a higher heart rate, has been shown to be effective in shortening QT intervals and terminating torsade de pointes (TdP) (4). However, overdrive pacing in the acute management of osimertinib-induced VAs has rarely been reported in the literature. Here, we report a case of osimertinib-induced QT prolongation, frequent VAs, and TdP for which a temporary pacemaker and overdrive pacing were used. Serial electrocardiography (ECG) and Holter monitoring results during hospitalization and follow-ups confirmed the in-hospital and long-term efficacy and safety of these treatments.

## Case presentation

**Figure 1:**
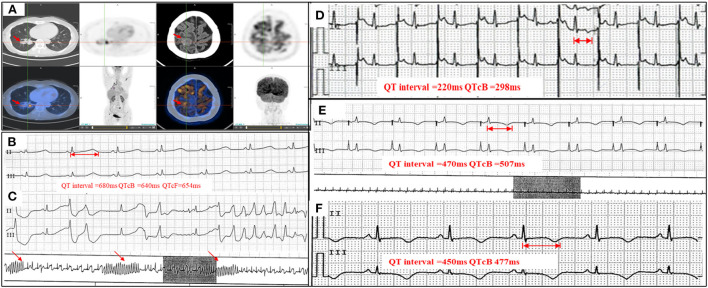
PET-CT, serial Holter monitoring, and ECG. **(A)** PET-CT showing peripheral pulmonary carcinoma and brain metastasis (arrowheads); **(B)** Holter monitoring on admission showing prolonged QTc interval (QTcB 640 ms); **(C)** Holter monitoring on admission showing frequent VTs and TdP (arrowheads); **(D)** ECG with a temporary pacemaker and overdrive pacing at 110 bpm (QTcB 298 ms); **(E)** Holter monitoring with a temporary pacemaker and pacing at 70 bpm (QTcB 507 ms); **(F)** ECG at discharge showing near-normal QTc interval (QTcB 477 ms). VT, ventricular tachycardia; TdP, torsade de pointes; QTcB, QTc interval calculated with Bazett formula; PET-CT, positron emission tomography – computed tomography; ECG, electrocardiogram.

A 60-year-old woman was admitted to our hospital with palpitations and an onset of syncope. The patient had experienced palpitations 3 months previously while working and one episode of syncope later at home. The patient had regained consciousness after 10 seconds but took no action and sought no treatment. Two days preceding admission, the palpitations had become more frequent, and the patient reported feeling dizzy on several occasions. The symptoms were not related to exercise or emotional changes. Seventeen months previously, the patient had been diagnosed with peripheral lung adenocarcinoma and associated brain and bone metastases (Figure 1A). Genomic analysis had indicated *EGFR* gene mutations, and she had therefore been treated with the EGFR TKI osimertinib (80 mg, QD). The patient had no history of hypertension, diabetes or related family history. There was also no record of previous use of anti-arrhythmic agents.

The patient presented with tachycardia (122 bpm) and hypotension (81/68 mmHg) on physical examination. Electrocardiography (ECG) on admission showed prolonged QTc intervals (QTcB 577 ms) and frequent ventricular premature complexes (VPCs). Holter analysis also showed prolonged QTc intervals (longest QTcB 640 ms, Figure 1B), frequent VPCs, and ventricular tachycardias (VTs) (122 episodes/20 h, including multiple TdP) (Figure 1C). Echocardiography showed no structural or functional abnormalities. Laboratory assessments showed normal electrolyte concentrations (K^+^ 3.9 mmol/L, Ca^2+^ 2.50 mmol/L, Mg^2+^ 0.93 mmol/L) and negative cardiac biomarkers. SCN5A and KCH2 mutations were not detected during genetic screening. An ECG that had been conducted prior to osimertinib treatment showed normal QTc intervals (QTcB 417 ms, Supplementary Figure 1A). After ruling out QT prolongation caused by myocardial ischemia or other QT-prolongation drugs, the patient was diagnosed with osimertinib-induced QT prolongation, VAs, and TdP (Probable causality, World Health Organization-Uppsala Monitoring Center [WHO-UMC] causality assessment scale).

Osimertinib was immediately discontinued after it was identified as the etiology for QT prolongation and VAs. Anti-arrhythmia treatments (intravenous magnesium, potassium magnesium aspartate, and oral propranolol) were administered; however, they did not relieve the patient's symptoms. No defibrillation treatment was delivered for a stable hemodynamic status.

After a temporary pacemaker was implanted with the pacing lead placed at the patient's right atrium, overdrive pacing successfully shortened the QTc interval and terminated the VAs. The initial pacing rate of 110 bpm was gradually reduced to 60-−70 bpm within 1 week, and the QTc interval was shortened to 298–507 msec (Figures 1D,E). Repeat Holter monitoring at 1 week showed no VPCs or VTs. The pacemaker was then removed and following consultation with a hematologist, osimertinib was replaced by gefitinib (250 mg QD). At discharge, the patient's symptoms were relieved and her ECG showed normal results (QTc 477 ms) (Figure 1F, Supplementary Figure 1B). Routine ECG surveillance was conducted. Three- (Supplementary Figure 1C) and 6-month follow-ups showed good recovery and normal ECG results (Table 1).

**Table 1:** Time line.

17 months prior to presentation	• Diagnosed with peripheral lung adenocarcinoma with associated brain and bone metastases. • Osimertinib treatment started.
3 months prior to presentation	• Palpitations whilst working. • One episode of syncope at home.
2 days prior to presentation	• Frequent palpations, dizziness.
At presentation	• BP 81/68 mmHg, BMI 14.9 kg/m^2^ (164 cm/40 kg) • ECG: HR 122 bpm, QTcB 532 msec, frequent VPCs. • Normal electrolyte concentrations. • Negative cardiac biomarkers. • Anti-arrhythmias medical treatment: including intravenous magnesium, potassium magnesium aspartate and lidocaine, and oral propranolol. • Osimertinib was discontinued.
2 days later	• Symptoms not relieved. • Holter monitoring: prolonged QTc interval (longest QTcB 640 ms), frequent VPCs and VTs (122 episodes/20 h, including multiple TdP) • Treatment: temporary pacemaker implantation. Overdrive pacing (at 110 bpm) shortened the QTc interval and terminated VAs. QTcB 298 ms.
9 days later	• With temporary pacemaker, the pacing rate was gradually reduced to 60–70 bpm; ECG: HR 70 bpm, QTcB 507 msec. • No palpitation and syncope. Holter monitoring: no VAs. Temporary pacemaker removed.
15 days later	• ECG: HR 67 bpm, QTcB 477 ms • No palpitation and syncope
3 months later	• No palpitation and syncope, good recovery • ECG: HR 68 bpm, QTcB 479 ms
6 months later	• No palpitation and syncope, good recovery

BP, Blood pressure; HR, Heart rate; BMI, Body mass index; ECG, Electrocardiograph; QTcB, Corrected QT interval by Bazett formula; TdP, Torsade de pointes; VPCs, Ventricular premature complexes; VT, Ventricular tachycardias; VAs, Ventricular arrhythmias.

## Discussion

This study reported a case of osimertinib-induced QT prolongation accompanied by frequent VAs and TdP in a patient being treated for NSCLC.

EGFR mutation is one of the most common oncogenic drivers in NSCLC. As such, EGFR-TKIs (including gefitinib, erlotinib, and osimertinib, etc.) are used to inhibit EGFR tyrosine kinase and have enhanced the treatment for NSCLC over the past two decades (5). In particular, osimertinib, a third-generation EGFR-TKI, has been shown to increase treatment efficacy even when compared with that of first- or second-generation EGFR TKIs (6). Osimertinib has thus become the first-line treatment for advanced EGFR-mutant NSCLC patients, especially for those with brain metastases or acquired T790M resistance mutation (1).

Despite their low incidence, cardiotoxicities including congestive heart failure, QT prolongation, and vital arrhythmias have become a safety concern for patients taking EGFR TKIs. Osimertinib-induced QT prolongation was first reported during the phase I trials for the drug (7), after which analyses in two phase III randomized controlled trials also confirmed that osimertinib notably increased the risk of cardiac toxicities, with a risk ratio of 2.62 for QT prolongation (8). The initial FDA risk-benefit assessment reported a low incidence (0.7%) of osimertinib-induced substantial QTc prolongation (QTc ≥ 500 msec), with no QTc-related VAs reported (9). Further, when Anand et al. reviewed the pharmacovigilance database of the FDA Adverse Events Reporting System (FAERS), and compared the cardiotoxicities of different EGFR-TKIs, a total of 315 cardiac adverse events (AE) were noted. Cardiac failure and QT prolongation were the cardiotoxicities most commonly caused by osimertinib. Of patients treated with osimertinib, 33/2,454 (1.3%) developed QT prolongation at a median time of 23 days. A comparison with first- and second-generation EGFR-TKIs has shown that osimertinib is more likely than the others to induce QT prolongation (reported odds ratio 6.6) (2). In a recent retrospective cohort study, Kunimasa et al. compared QT intervals in 72 patients with serial ECGs before and after osimertinib administration and found that QTc intervals were prolonged by approximately 20 ms over a median time of 116 days. However, no fatal arrhythmias were reported in this study (10).

In addition to QT prolongation, VT or TdP were also reported in a limited number of cases taking osimertinib (Table 2) (11–14). This indicates that osimertinib-induced vital arrhythmias are probably underestimated owing to the limitations of retrospective studies and the reporting system. Physicians should be vigilant to the occurrence of these rare vital arrhythmias in patients on osimertinib and conduct ECG surveillance for these patients.

**Table 2:** Summary of osimertinib-induced VAs cases reported.

**Author [Ref]**	**Osimertinib treatment time (month)**	**QTc interval (ms)**	**Type of VAs**	**Anti-arrhythmias treatment**	**Follow-up**
Matsuura et al. (11)	2	486	TdP	• Osimertinib discontinued; • Magnesium supplementation.	• Not mentioned
Ikebe et al. (12)	2	524	TdP	• Osimertinib discontinued; • Cardioversion;	• Died of cancer progression and cachexia 15 months after osimertinib discontinuation.
Bian et al. (13)	6	647	TdP	• Osimertinib discontinued; • Magnesium supplementation, potassium supplementation, and administration of antiarrhythmic drug lidocaine.	• QT interval got closer to normal gradually, but the patient experienced decreased blood pressure, pulse oxygen saturation, and was unconscious. In order to relieve the patient's pain, the patient was discharged without invasive salvage measures.
Kaira et al. (14)	3	>600	VF, Cardiac arrest	• Osimertinib discontinued; • Cardiovascular agents (not specified). • Cardiopulmonary resuscitation.	• Not mentioned.
Our case	17	640	VT, TdP	• Osimertinib discontinued • Intravenous magnesium, potassium magnesium aspartate, and oral propranolol; • Overdrive pacing by temporary pacemaker	• Osimertinib was replaced by gefitinib (250 mg QD). • QT interval getting closer to normal gradually, the three- and 6-month follow-ups showed good recovery and normal ECG results.

TdP, Torsade de pointes; VT, Ventricular tachycardias; VAs, Ventricular arrhythmias.

However, the mechanism of osimertinib-induced cardiotoxicity is still unclear (15). In the preliminary IC50 inhibition *in-vitro* cell test, osimertinib showed weak inhibition of the cardiac potassium channel Kv11.1, which may be a potential mechanism of osimertinib-induced QT prolongation (16). However, further basic research is required for full clarification of the underlying mechanism.

As the treatment of VTs is based on the determination of their etiology, owing to the lack of understanding of the mechanism behind osimertinib-related arrhythmias, there has been no consensus for appropriate management. Magnesium supplementation, cardioversion, and β-blockers are generally used in the management of long QT syndrome (LQTS) related VT and Tdp (4). In the limited cases of osimertinib-induced VAs, point-of-care monitoring-guided magnesium supplementation, cardioversion, and antiarrhythmic drugs have been reportedly used (Table 2) (11–14); however, in this case, all treatment failed to improve this patient's symptoms. Although implantable cardioverter defibrillator (ICD) implantation is suggested in high-risk LQTS patients, it would have certainly led to frequent shocks for this particular patient, and was therefore deemed unsuitable. Additionally, the presence of polymorphic VT and TdP, indicated the ineligibility for radiofrequency ablation. Lastly, left cardiac sympathetic denervation (LCSD) is regarded as a bail-out strategy in the case that other treatments should fail.

Medically (isoprenaline infusion) or electrically (override pacing) speed up the heart can both help to decrease the QTc interval and terminate TdP temporarily. The efficacy of override pacing in comparison with isoprenaline is uncertain due to the lack of randomized comparison evidence (17). However, override pacing would be a better option when the risk of TdP may persist over a more extended period, such as a long-acting drug. As the mean elimination half-life time of osimertinib is 48–59.7 h theoretically (18, 19), temporary pacemaker implantation and overdrive pacing can help to shorten the QTc interval and increase survival during this life-threatening time period.

On the other hand, osimertinib was replaced with gefitinib for chemotherapy after the occurrence of this life-threatening complication. No disease progression or TdP recurrence has been detected and favorable recovery has been archived in the follow-ups.

## Conclusions

Osimertinib-induced QT interval prolongation and VAs are underestimated in NSCLC patients, and no consensus has been achieved on standard treatment. This case showed that ECG and Holter monitoring should be performed periodically in patients on osimertinib treatment. Temporary pacemaker implantation and overdrive pacing may be considered a safe and effective treatment for the acute management of osimertinib-induced VAs.

## Data availability statement

The original contributions presented in the study are included in the article/Supplementary material, further inquiries can be directed to the corresponding author/s.

## Ethics statement

Written informed consent was obtained from the participant for the publication of this case report.

## Author contributions

YZ and BD identified the case. XW and YP conducted the literature search and prepared the first draft of the manuscript. KN and PY provided critical revision for the manuscript. All authors contributed to the articles and approved the submitted version.

## Funding

This research was supported by grants from Jilin Provincial Science and Technology Department International Cooperation Project (No. 20210402016GH) and the National Natural Science Foundation of China (No. 82100337).

## Conflict of interest

The authors declare that the research was conducted in the absence of any commercial or financial relationships that could be construed as a potential conflict of interest.

## Publisher's note

All claims expressed in this article are solely those of the authors and do not necessarily represent those of their affiliated organizations, or those of the publisher, the editors and the reviewers. Any product that may be evaluated in this article, or claim that may be made by its manufacturer, is not guaranteed or endorsed by the publisher.

## Supplementary material

Supplementary Figure 1 Serial 12 leads ECGs before osimertinib treatment and in the recovery stage. **(A)** 12 leads ECG four months before osimertinib treatment showing normal QTc interval (QTcB 417 ms); **(B)** 12 leads ECG at discharge showing near-normal QTc interval (QTcB 477 ms); **(C)** 12 leads ECG at three month's follow-up showing near-normal QTc interval (QTcB 468 ms); QTcB, QTc interval calculated with Bazett formula.
