# Supplementary figures and images for: Overdrive pacing in the acute management of osimertinib-induced ventricular arrhythmias: A case report and literature review
Source: Front Cardiovasc Med. 2022 Sep 29;9:934214. doi: 10.3389/fcvm.2022.934214 (PMC9557095; doi:10.3389/fcvm.2022.934214)

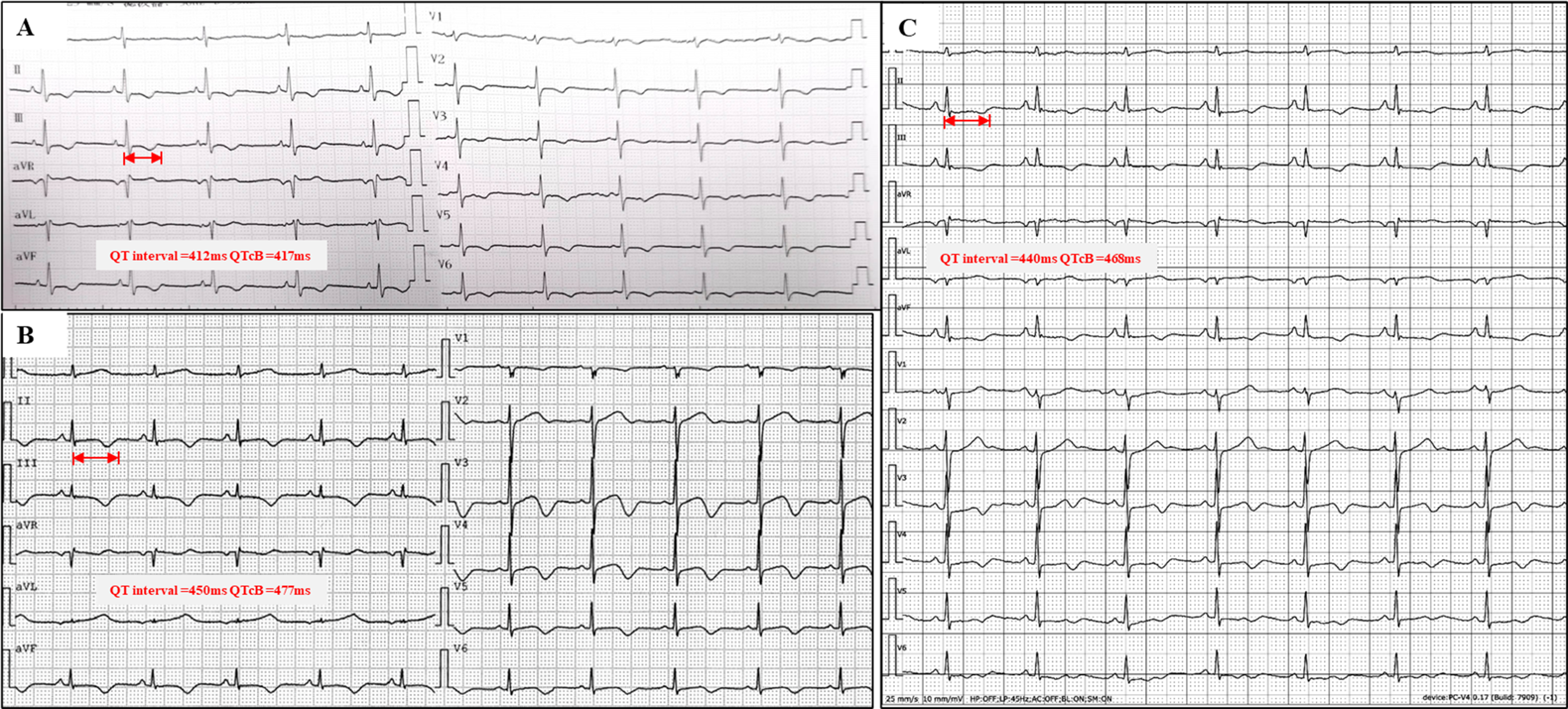

Supplement: Supplementary Figure 1 — Serial 12 leads ECGs before osimertinib treatment and in the recovery stage. (A) 12 leads ECG four months before osimertinib treatment showing normal QTc interval (QTcB 417 ms); (B) 12 leads ECG at discharge showing near-normal QTc interval (QTcB 477 ms); (C) 12 leads ECG at three month's follow-up showing near-normal QTc interval (QTcB 468 ms); QTcB, QTc interval calculated with Bazett formula. [file Image_1.TIF]
